# A machine learning approach for the prediction of pulmonary hypertension

**DOI:** 10.1371/journal.pone.0224453

**Published:** 2019-10-25

**Authors:** Andreas Leha, Kristian Hellenkamp, Bernhard Unsöld, Sitali Mushemi-Blake, Ajay M. Shah, Gerd Hasenfuß, Tim Seidler

**Affiliations:** 1 Department of Medical Statistics, University Medical Center Göttingen, Göttingen, Germany; 2 Clinic for Cardiology and Pulmonology/Heart Center, University Medical Center Göttingen, Göttingen, Germany; 3 Department of Internal Medicine II, University of Regensburg, Regensburg, Germany; 4 King’s College London British Heart Foundation Centre, School of Cardiovascular Medicine & Sciences, London, England, United Kingdom; 5 DZHK (German Centre for Cardiovascular Research), Partner Site Göttingen, Göttingen, Germany; Wayne State University, UNITED STATES

## Abstract

**Background:**

Machine learning (ML) is a powerful tool for identifying and structuring several informative variables for predictive tasks. Here, we investigated how ML algorithms may assist in echocardiographic pulmonary hypertension (PH) prediction, where current guidelines recommend integrating several echocardiographic parameters.

**Methods:**

In our database of 90 patients with invasively determined pulmonary artery pressure (PAP) with corresponding echocardiographic estimations of PAP obtained within 24 hours, we trained and applied five ML algorithms (random forest of classification trees, random forest of regression trees, lasso penalized logistic regression, boosted classification trees, support vector machines) using a 10 times 3-fold cross-validation (CV) scheme.

**Results:**

ML algorithms achieved high prediction accuracies: support vector machines (AUC 0.83; 95% CI 0.73–0.93), boosted classification trees (AUC 0.80; 95% CI 0.68–0.92), lasso penalized logistic regression (AUC 0.78; 95% CI 0.67–0.89), random forest of classification trees (AUC 0.85; 95% CI 0.75–0.95), random forest of regression trees (AUC 0.87; 95% CI 0.78–0.96). In contrast to the best of several conventional formulae (by Aduen et al.), this ML algorithm is based on several echocardiographic signs and feature selection, with estimated right atrial pressure (RAP) being of minor importance.

**Conclusions:**

Using ML, we were able to predict pulmonary hypertension based on a broader set of echocardiographic data with little reliance on estimated RAP compared to an existing formula with non-inferior performance. With the conceptual advantages of a broader and unbiased selection and weighting of data our ML approach is suited for high level assistance in PH prediction.

## Introduction

Within the broader field of artificial intelligence science, the term machine learning (ML) refers to advanced algorithms with features of supervised or even unsupervised adoption to problem solving [1,2]. Such algorithms need to be "trained" with data that is annotated to a variable of interest in order to give a mathematical model for more general applicability. This model is then capable of generalization to solve annotation tasks on similar data of unknown annotation. While this concept has existed for many decades, recent conceptual advances and a significant increase in computational capacity suggest ML may be on the verge of becoming a valuable clinical tool [3–5]. Frequently, clinical tasks require integration of a multitude of variables to be weighted for estimation of the likelihood of a diagnosis or outcomes. ML-assisted decision making should theoretically be advantageous over decisions based on experience or reasoning alone due to its capacity to process information with less bias as well as measurable, comparable and constant performance [6]. However, the application of more advanced ML algorithms to assist in cardiovascular diagnostics is only emerging [7]. In cardiology, echocardiographic estimation of the likelihood of a diagnosis may be ideally suited for a ML assisted approach, as a large amount of data from an individual needs to be integrated intellectually by the examiner. Moreover, computational data processing is already an integrated technical part of the echocardiographic examination facilitating its early adoption [8,9].

Mean pulmonary artery pressure (PAPm) ≥ 25mmHg measured by right heart catheterization (RHC) defines pulmonary hypertension (PH). Echocardiographic estimation of the likelihood of pulmonary hypertension is an important clinical problem, as it is required to establish sufficient pre-test probability to control risks and resources of the invasive RHC examination. To estimate the likelihood of PH it is possible to achieve an approximation of PAP using echocardiography. This is conducted by calculating the pressure difference between RA and RV from tricuspid regurgitation velocity (TRV) (using the simplified Bernoulli equation) and by adding right atrial pressure (RAP) to this value. However, the European guidelines recommend considering TRV instead of estimating PAP. This is due to concerns that derived values, in particular estimation of RAP, exaggerate error. Instead, estimation of the likelihood is based on consideration of categorical values of TRVmax (cutoff 2.8 m/s and 3.4 m/s) and the presence of any one additional sign for PH from a set of several echocardiographic signs. Although we have recently demonstrated that PH can be predicted with high accuracy based on echocardiographically estimated RAP/PAP in the particular setting of experienced examiners, we also endorse the restrictive recommendation regarding estimating RAP as mentioned in the guidelines but in view of a more general applicability. Therefore, the approach to allow for consideration of a broad set of echocardiographic signs with less emphasis on RAP seems more robust. Unfortunately, there is no systematic scientific evaluation of the suggested guideline approach. Thus, we sought to establish an algorithm for echocardiographic prediction of PH, that 1) is based on ML to ensure its objective unbiased generation, 2) includes a relatively broad, yet routinely obtained set of parameters in order to achieve sensitivity and limit reliance on very few and problematic parameters such as RAP estimation, while avoiding lengthy or complicated examinations 3) yields meaningful weights of informative vs. less informative signs and 4) achieves the same or a higher level of sensitivity while retaining overall predictive performance for the presence of PH (i.e. similar AUC in ROC analysis) compared to the currently best performing algorithm for estimating PAP in experienced hands. We present the development and internal validation of a machine learning model to diagnose PH from echocardiographic measurements.

## Methods

An expanded methods section is included in the online supplement (S1 Appendix).

### Study population

This paper presents results obtained on data from a retrospective study on echocardiographic examinations and the results of RHC performed at the Clinic for Cardiology and Pulmonology, University Medical Center Göttingen; King’s College Hospital, London; and the Department of Internal Medicine II, University of Regensburg between 2011 and 2016 [10]. The study was conducted as a database search limited to echocardiographic and RHC data as approved by the local ethics committees and in accordance to the amended Declaration of Helsinki. All data were fully anonymized before the data were accessed. Inclusion criteria were (1.) invasively determined pulmonary artery pressure (PAP) within 24 hours after echocardiographic examination and (2.) sufficient data quality defined as at most 40% of the relevant information missing.

### Risk factor variables

As risk factor variables the basic patient characteristics of age, gender, BMI, and body surface area (BSA) were used in conjunction with 21 echocardiographic measurements. RVD variables are defined as detailed in Rudski et al. [11]. The variable set especially allows calculation of the risk of PH using several established methods. Most variables show some missing values (Table 1). No variables were dropped due to missing values. To obtain unbiased performance estimates from the cross validation (CV) no pre-imputation was performed, but handling of missing values was conducted as part of the CV.

**Table 1:** Characterization of patients.

parameter	level	noPH	PH	p value
n		22	68	
age (years)				**< 0.01**
	mean ± sd	54 ± 19	68 ± 14	
	median(min; max)	55(21; 83)	74(22; 88)	
	missing	0	0	
sex				1.0
	m	8(50.0%)	32(47.1%)	
	w	8(50.0%)	36(52.9%)	
	missing	6	0	
BMI (kg/m^2^)				0.15
	mean ±sd	25 ±3.6	26 ±3.9	
	median (min; max)	26(18;29)	26 (19;38)	
	missing	7	2	
BSA (m^2^)				0.3
	mean ±sd	1.9 ±0.26	1.9 ±0.23	
	median(min;max)	2(1.4;2.3)	1.9(1.4;2.5)	
	missing	7	2	
IVSd (mm)				0.7
	mean ±sd	12 ±3.3	12 ±2.7	
	median (min; max)	12(8;22)	12(7;21)	
	missing	6	3	
LVEDD (mm)				0.25
	mean ±sd	46 ±8.2	49 ±12	
	median (min;max)	46(29;62)	47(26;78)	
	missing	6	3	
PW (mm)				0.81
	mean ±sd	12 ±3.2	12 ±2.8	
	median (min;max)	12 (7;22)	12 (8;24)	
	missing	6	3	
LAD (mm)				0.59
	mean ±sd	41 ±9.7	43 ±10	
	median (min; max)	40 (29;55)	46 (23;68)	
	missing	16	35	
EF (%)				0.12
	mean ±sd	52 ±17	46 ±14	
	median (min; max)	55 (10;80)	53 (10;66)	
	missing	0	3	
RVD1 (mm)				**0.02**
	mean ±sd	41 ±7.6	48 ±8.7	
	median (min; max)	42 (25;51)	49 (26;66)	
	missing	10	18	
RVD2 (mm)				**< 0.01**
	mean ±sd	31 ±4.8	37 ±9.6	
	median (min; max)	31 (22;38)	36 (18;63)	
	missing	10	18	
RVD3 (mm)				0.36
	mean ±sd	72 ±9.7	75 ±12	
	median (min; max)	72 (58;90)	74 (48;100)	
	missing	10	21	
RVenlargement (0/1)				0.72
	0	4 (33.3%)	13 (26.0%)	
	1	8 (66.7%)	37 (74.0%)	
	missing	10	18	
TAPSE (mm)				**0.02**
	mean ± sd	21 ±5.2	17 ±4.6	
	median (min; max)	22 (12;33)	17 (8;29)	
	missing	6	11	
RAPestimated (mmHg)				**< 0.01**
	mean ±sd	5.4 ±3.2	8.9 ±5.4	
	median (min; max)	3 (3;15)	8 (3;15)	
	missing	0	0	
RAP≥15 (0/1)				**< 0.01**
	0	21 (95.5%)	41 (60.3%)	
	1	1 (4.5%)	27 (39.7%)	
	missing	0	0	
AS (0/1/2/3)				0.73
	mean ±sd	0.47 ±0.99	0.33 ±0.81	
	median (min; max)	0 (0;3)	0 (0;3)	
	missing	7	2	
AR (0/1/2/3)				0.38
	mean ±sd	0.4 ±0.74	0.39 ±0.6	
	median (min; max)	0 (0;2)	0 (0;2)	
	missing	7	2	
MS (0/1/2/3)				0.32
	mean ± sd	0 ±0	0.17 ±0.45	
	median (min; max)	0 (0;0)	0 (0;2)	
	missing	7	2	
MR (0/1/2/3)				0.85
	mean ± sd	0.87 ±0.99	1.1 ±0.98	
	median (min; max)	1 (0;3)	1 (0;3)	
	missing	7	1	
TR (0/1/2/3)				0.21
	mean ±sd	1.3 ±1.1	1.7 ±0.89	
	median (min; max)	1 (0;3)	2 (0;3)	
	missing	7	2	
TRVmax (m/s)				**< 0.01**
	mean ±sd	2.7 ±0.6	3.4 ±0.84	
	median (min; max)	2.6 (1.8;3.9)	3.3 (1.6;5.5)	
	missing	1	2	
TRVm (m/s)				**< 0.01**
	mean ±sd	1.9 ±0.43	2.5 ±0.59	
	median (min; max)	1.9 (1.3;2.9)	2.4 (1.3;3.9)	
	missing	1	2	
PVAT (ms)				0.1
	mean ±sd	102 ±26	87 ±21	
	median (min; max)	100 (60;150)	83 (56;141)	
	missing	11	33	
TRPm (mmHg)				**< 0.01**
	mean ±sd	15 ±6.8	26 ±12	
	median (min; max)	14 (6.5;32)	23 (6.6;61)	
	missing	1	2	
WHO classification				
	0: no PH	21 (95.5%)	0 (0.0%)	
	1: PAH	0 (0.0%)	6 (8.8%)	
	2: due to LH-Disease	1 (4.5%)	48 (70.6%)	
	3: due to lung diseae	0 (0.0%)	2 (2.9%)	
	4: CTEPH	0 (0.0%)	0 (0.0%)	
	5: unknown / multifactorial	0 (0.0%)	12 (17.6%)	
Incident Case				
	0: known PH or pre-evaluated patient	0 (0.0%)	13 (19.1%)	
	1: new evaluation	22 (100.0%)	55 (80.9%)	

The cohort of the available 90 patients grouped into 68 patients with confirmed (by means of RHC) PH and 22 patients without PH. This table shows descriptive values for four basic characteristics age, sex, BMI and body surface area (BSA) as well as for 23 echocardiographic measurements and the WHO classification. The last column contains p values from comparisons between the two patient subgroups. t test and χ2 test were used as appropriate.

### Outcomes

Presence or absence of PH was the pre-defined outcome of this analysis and following the 2015 European Society of Cardiology guidelines for the diagnosis and treatment of PH was defined as PAPm ≥ 25 mm Hg as assessed at rest by RHC [12]. For regression methods direct modeling of the PAPm measurement itself is used as alternative.

### Machine learning algorithms

Five ML algorithms were evaluated: support vector machine (SVM [13]) lasso penalized logistic regression [14], boosted classification tree models using Quinlan's C5.0 algorithm [15], random forest of classification trees, and random forest of regression trees [16]. Technical details are given in the supplement (S1 Appendix). The guidelines of the transparent reporting of a multivariable prediction model for individual prognosis or diagnosis (TRIPOD) statement were followed (S1 Appendix).

### Statistical analysis

Descriptive values were computed for all variables under consideration. Factor analysis for mixed data (FAMD [17]) was used to extract components explaining most of the variance. Variables with established cutoffs for dichotomization into high and low were dichotomized for the machine learning evaluation.A 10 times repeated 3-fold CV leading to 3 folds of size 28 in each repetition was constructed (S3 Fig). The ML algorithms were then trained in turn on two partitions and evaluated on the remaining partition. The low number of folds was chosen in order to arrive at large test sets which allow for reasonable imputation within each fold. A stratified sampling scheme was applied to achieve the same distribution in all training and test sets. Within the CV both training and test set were imputed separately so as to avoid a bias in the performance estimation.

The random forest methods as well as the boosted classification trees are able to handle missing values internally. Prior to applying the other algorithms, missing values were imputed using the iterative FAMD algorithm [18]. Reported are performance measures, especially the area under the receiver operator characteristic (ROC) curve (AUC), averaged over the 10 repetitions. Confidence intervals for the AUC were calculated from the CV using the method by LeDell et al. [19] specifically dealing with the CV structure. Variable importance for the regression tree forest was calculated using Breiman-Cutler permutation variable importance (16). All analyses were performed using the statistical programming environment R (version 3.4.3, [20]).

## Results

### Study population characteristics

The data set comprised 90 patients of which 68 (75.6%) had invasively confirmed PH using the recommended criterion of PAPm ≥25 mm Hg and of which 22 (24.4%) did not exhibit PH in the invasive measurement. Six patients were dropped from the analysis due to the high degree of missing values. Patients with confirmed PH were significantly older than patients without (68 ± 14 vs 54 ± 19 years; p < 0.01). As expected, several of the echocardiographic measurements (RVD1, RVD2, TAPSE, RAP, TRVmax, TRVm, and TRPm) show a significant difference between the two patient groups (Table 1).

The variables TRVmax, TRVm, and TRPm form a group of highly correlated variables (correlation coefficient between 0.94 and 0.99). The RVD variables (RVD1, RVD2, RVD3, and RVD enlargement) form another group of positively correlated variables; although the correlation to RVD3 is not strong enough to stay significant after correction for multiple testing. Both groups are slightly positively correlated with RVD2 showing the strongest correlation signal to the first group. All pairwise correlations have been calculated and variables were clustered (S1 and S2 Figs).

**Fig 1:**
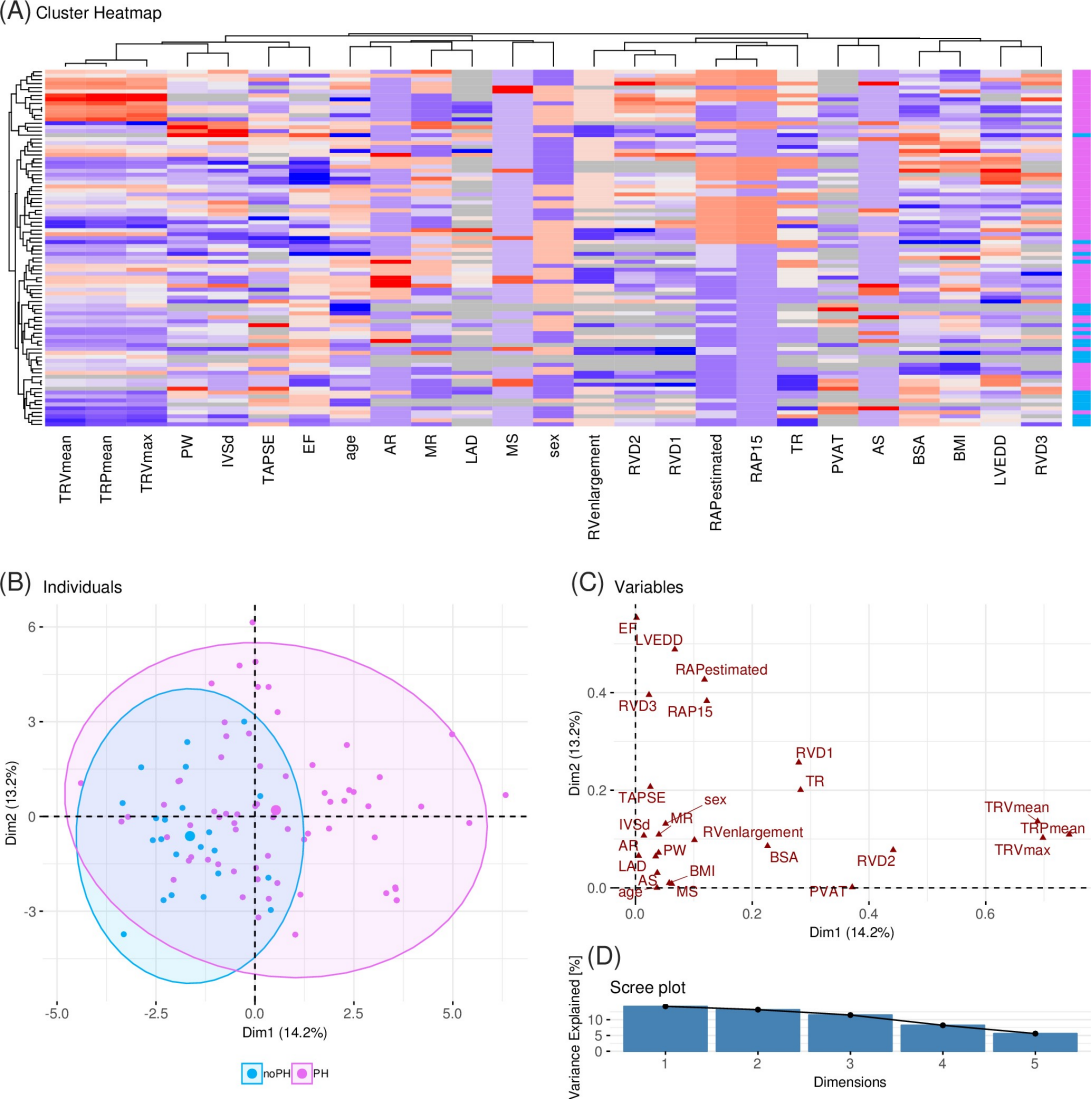
Data overview. (A) Heatmap of the 27 variables (columns) across the 90 patients (rows). The variables are studentized. Both patients and variables are re-ordered by hierarchical clustering. The color bar at the right shows patients with PH (pink) and without PH (blue). (B)-(D) Results from a factor analysis for mixed data (FAMD). (B) The first two dimensions explaining the largest parts of the variance in the data. Each dot represents one patient, where patients with PH are shown in pink, patients without confirmed PH are shown in blue. (C) Contribution of each of the variables to the first two dimensions of the FAMD. (D) Percentage of variance for the first five dimensions in the FAMD.

Correlation based clustering (Fig 1A) and factor analysis for mixed data (FAMD) (Fig 1B–1D) show some separation between patients with confirmed PH and patients without confirmed PH. The first dimension in the FAMD explains 15.6% of the variance and carries some separating tendency. The strongest signal towards this separation is due to the variables TRVm, TRVmax, and TRPm (Fig 1C).

### Prediction accuracy

**Fig 2:**
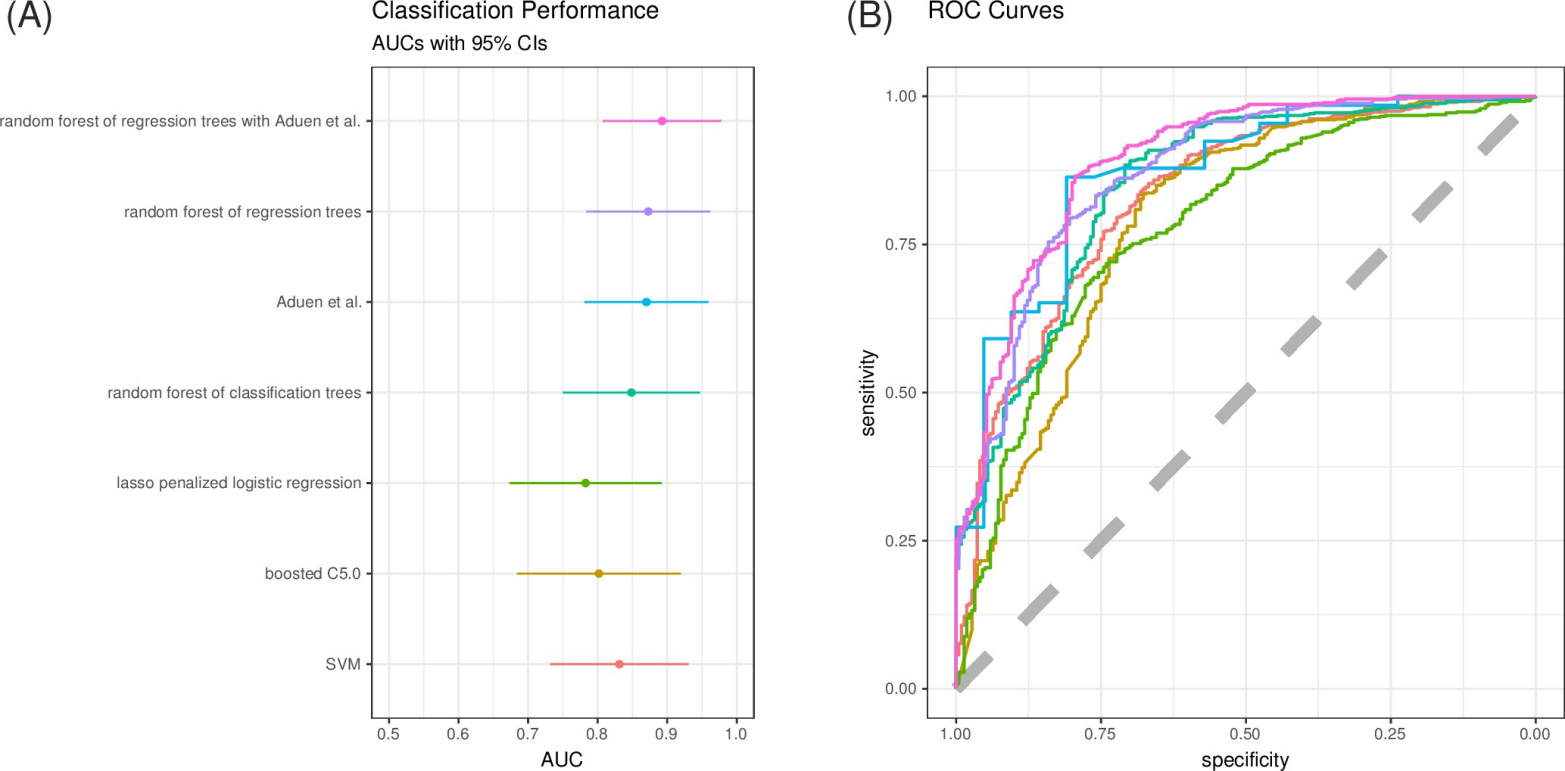
Classification performance. **Random forest of regression trees shows performance comparable to the best of several established PH prediction methods by Aduen et al.** (A) Area under the ROC curve (AUC) for all methods with estimated 95% confidence intervals. (B) Consensus ROC curves of the five machine learning algorithms under consideration as well as the ROC curve of the method by Aduen et al. (light blue).

Prediction performance measures as assessed within the CV for the ML algorithms as well as the established formula by Aduen et al. [21] demonstrated high accuracy (Table 2). For the ML methods mean values across CV repeats are shown. All methods yield AUC values > 0.78 and all confidence intervals largely overlap. No algorithm performs significantly worse than Aduen et al. (smallest p value 0.08 for the logistic regression according to DeLong's significance test for difference in ROC curves). However, the classification methods group at slightly lower levels (AUC values 0.80–0.85) whereas the random forest of regression trees achieve similar classification performance to the performance of Aduen et al. (AUC 0.87 in both cases). While Aduen et al. balances sensitivity (0.86) and specificity (0.86), the random forest of regression trees emphasizes sensitivity (0.89) over specificity (0.67) (Fig 2; for individual ROC curves S4 Fig). This emphasis of sensitivity over specificity is present for all trained machine learning methods and is due to the imbalance present in the studied cohort. Precision recall curves for the prediction of PH give a similar picture (S5 Fig). The combination of Aduen et al. with the random forest of regression trees achieves a slightly larger AUC of 0.89 (95% CI 0.81–0.98). Interestingly the emphasis of sensitivity (0.95) over specificity (0.52) is bigger still.

**Table 2:** Prediction performance measures.

method	AUC	ACC	sensitivity	specificity	PPV	NPV
Aduen et al.	0.87 [0.78; 0.96]	0.85	0.86	0.86	0.93	0.65
random forest of regression trees with Aduen et al.	0.89 [0.81; 0.98]	0.84	0.95	0.52	0.87	0.87
random forest of regression trees	0.87 [0.78; 0.96]	0.83	0.89	0.67	0.89	0.66
random forest of classification trees	0.85 [0.75; 0.95]	0.85	0.9	0.67	0.9	0.7
lasso penalized logistic regression	0.78 [0.67; 0.89]	0.8	0.93	0.4	0.83	0.65
boosted C5.0	0.80 [0.68; 0.92]	0.82	0.9	0.58	0.87	0.64
SVM	0.83 [0.73; 0.93]	0.84	0.95	0.49	0.85	0.76

Prediction performance is assessed using a 10 times repeated 3-fold CV and is measured using the AUC. The first column gives the AUC for the ML algorithms under consideration as well as the established method by Aduen et al. together with the 95% confidence interval according to DeLong. At the Youden index the accuracy, sensitivity, positive predictive value, and negative predictive value are evaluated additionally.

**Fig 3:**
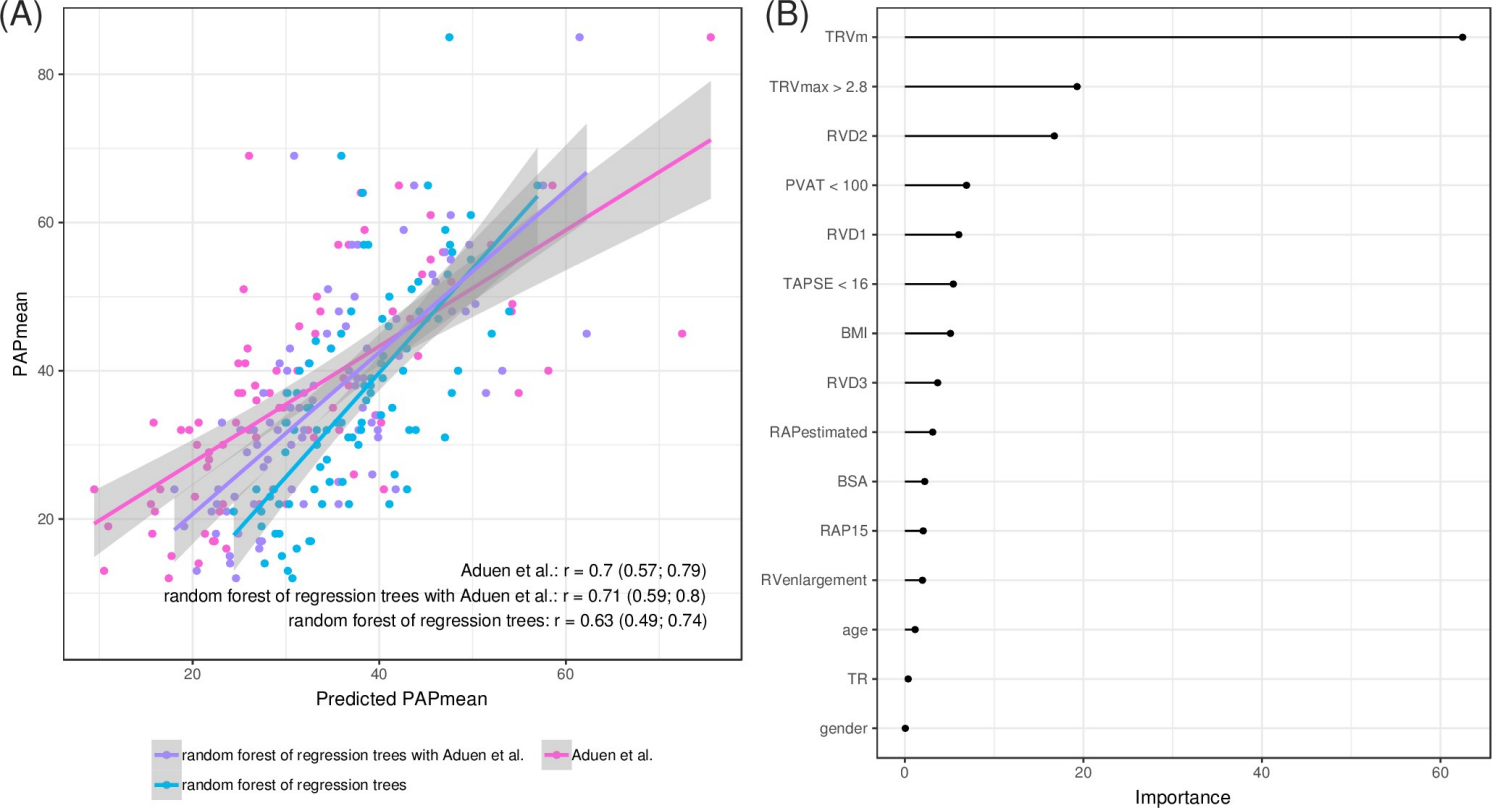
Best performing Machine Learning Method: Random forest of regression trees. The random forest of regression trees performed best among the 5 machine learning methods under consideration and achieves performance levels comparable to the prediction by Aduen et al., the best of several established prediction methods. (A) Invasively measured PAPm (y-axis) in comparison to the predictions (x-axis). Displayed are predictions by a random forest of regression trees (blue), predictions by the combination of the random forest of regression trees and the method of Aduen et al. (purple), and predictions by the method of Aduen et al. (pink). The lines show a linear fit with confidence bands (gray shades). The plot shows the predictions from the first repetition of the CI The text annotation gives Pearson's correlation coefficients with 95% confidence intervals. For the ML method these are average values across all CV repeats. (B) Variable importance for the random forest of regression trees.

The random forest of regression trees models the PAPm so that it is possible to compare the model predictions to the invasively measured values. The modeled values correlate with the invasively measured values at lower levels for the machine learning derived method compared to Aduen et al. with Pearson's correlation coefficients 0.63 for random forest and 0.70 for Aduen et al. The combined method achieves the same correlation again with a correlation coefficient of 0.69 (Fig 3A). The bias in the random forest of regression trees is smallest, while Aduen et al. on average underestimate the measured PAPm by 5mmHg. The random forest as well as the combined method, on the other hand, show regression to the mean behavior (S6 Fig).

### Machine-Learning variable rankings

Aduen et al. is calculated as the sum of TRPm and RAP and proved to be the best performing of several studied established prediction methods. Since the random forest of regression trees achieves comparable levels of classification performance, we asked which variables it predominantly uses. Permutation provides a manifest measure of the importance of variables within a random forest. For each variable the increase of the prediction error is averaged across all trees in the forest when the values of that variable are permuted. The RAP values show only little variable importance for the random forest of regression trees (Fig 3B). Instead the predictions are based mainly on TRVm, TRVmax and RVD2. TRVm is ranked as most important variable and RVD2 is among the most important variables also for all other algorithms except the lasso penalized logistic regression (S7 Fig).

## Discussion

**Fig 4:**
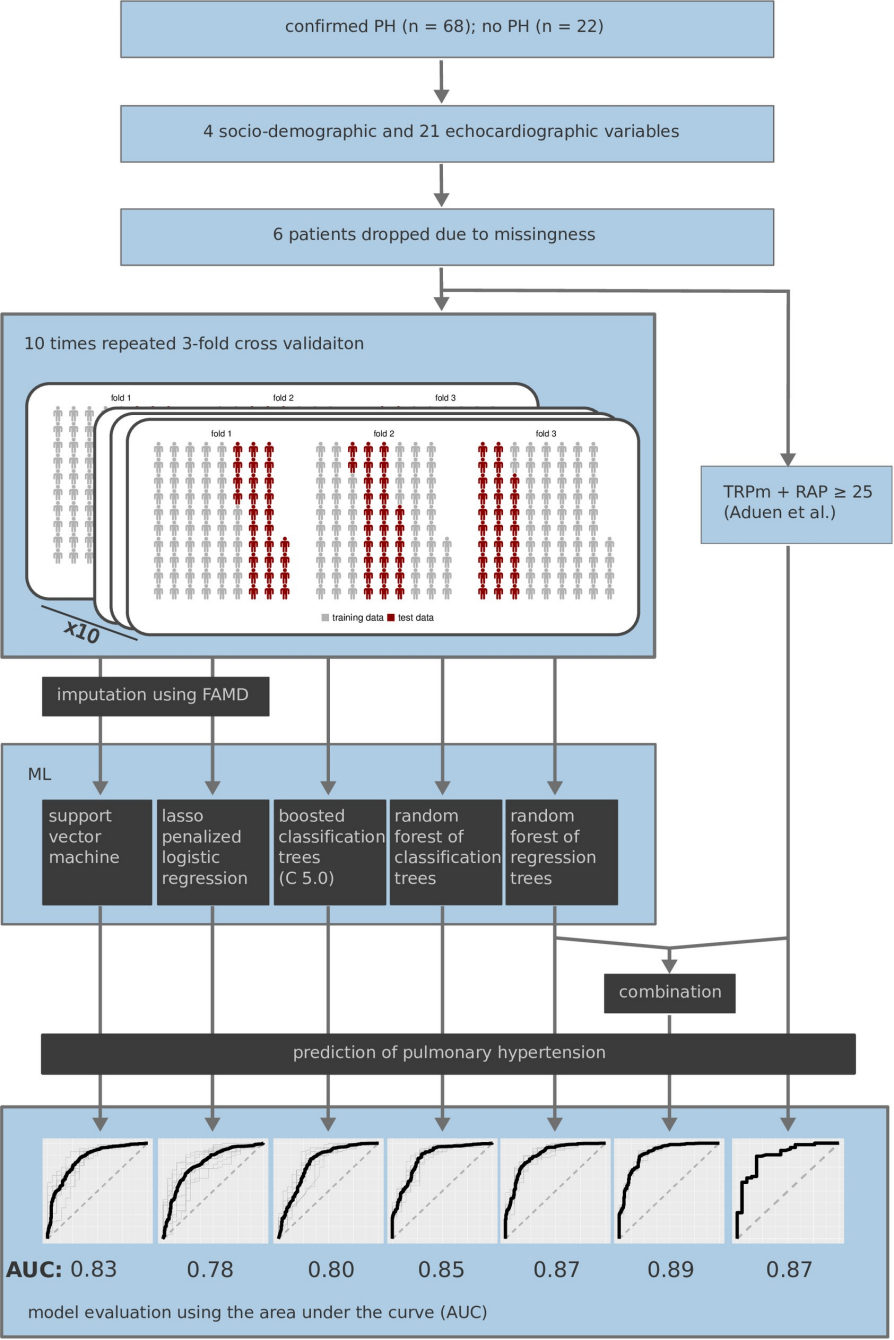
Overview procedure and main results. The data set comprises measurements of 68 patients with confirmed PH and 22 patients without PH. Four socio-demographic and 21 echocardiographic variables were measured. Six patients were dropped due to the high degree of missingness. As reference the formula by Aduen et al. was evaluated. Five ML methods were applied and evaluated using a 10 times repeated 3-fold CV scheme. Two ML methods required an imputation as pre-processing step within each fold of the CV. The predictions of the random forest of regression trees have additionally been combined with the predictions by Aduen et al.

Although only two echocardiographic measurements suffice to estimate PAP with high precision in experienced hands, the European guidelines prefer consideration of several signs over PAP due to concerns regarding error amplification [12]. To establish an algorithm that achieves this task, we interrogated several ML methods for their performance, as ML is ideally suited to integrate multiple parameters. Fig 4 gives an overview over the patient cohort, the collected data, and the processing (Fig 4). The large majority of the cohort were patients in WHO group 2 (due to left heart disease). A possible application of PH prediction in this cohort is risk estimation, e.g. in the context of interventional or surgical procedures.

Prediction of the likelihood of pulmonary hypertension based on echocardiography is a routine clinical task but in practice estimation of the likelihood and in particular PAP is still difficult, frequently resulting in inaccurate estimates and wrong diagnosis. In an earlier analysis we sought to identify the best of various existing algorithms and found the relatively simple formula first described by Aduen at al.: PAPm = TRPm + RAP performed best and with high accuracy in the particular setting of experienced examiners [10]. In this formula, RAP was informative, i.e. inclusion increased the predictive accuracy (as compared to TRPm alone). However, estimating RAP under routine conditions is not a trivial task and has been criticized in current guidelines for its potential to increase inaccuracy [12]. Moreover, due to its few parameters the formula might ignore several typical features of PH that can be readily uncovered by routine echocardiographic examination. This suggests that meaningful information may be lost and that the result is highly prone to erroneous measurement of one of the two parameters e.g. in less experienced hands. ML provides an unbiased approach to derive predictions with the potential to recognize unknown interactions and assist in meaningful feature selection. Machine learning is not principally more advantageous over experienced examiners who through experience integrate several parameters with high accuracy without statistical reasoning. Rather, it offers a contemporary solution to standardize and simplify consideration, integration and reasoning based on several parameters [22]. Thus, we examined ML for its capability to assist in assigning the likelihood of PH. With the intention to include ML in future echocardiographic devices, our aim was to address typical problems in addressing the likelihood of PH during routine echocardiography.

We found that: 1) the regression based random forest ML method identified patients with PH (confirmed by RHC within 24h) with very high accuracy. 2) Feature importance analysis demonstrated that this ML algorithm was largely independent of estimated RAP at the same time as being capable of integrating various echocardiographic features of PH and thus capable of managing missing values. 3) The studied binary classification methods achieve slightly (not significantly) lower discrimination levels (assessed by AUC). 4) Both traditional formula and ML algorithms may be combined to further increase sensitivity albeit at the expense of specificity.

RAP is considered an error-prone parameter and therefore not recommended by the ESC guidelines [12] due to its dynamic change depending on fluid intake, the requirement of a subcostal view that is robust towards deep respiration, a patient that complies with a breathing command and difficulties in measuring vena cava diameter during movement of the liver during inspiration. Although these issues can be addressed well in the majority of patients by experienced examiners, avoiding Aduen et al. may lead to an even more robust result in the setting of less experienced examiners. As we determined Aduen et al to represent the best performing established method in an earlier analysis, the dataset is limited to a set of patients were application of the method of Aduen et al. was possible. Therefore, the dataset is restricted to patients for whom a good subcostal view and compliance with respiratory commands for calculation of RAP were achieved. Hence, in this setting ML offers at least a powerful alternative to Aduen et al., by selecting additional parameters without loss of precision. Furthermore ML favorably addresses the desire to incorporate as much information as possible while allowing for the clinical reality of some missing values. Due to these conceptual advantages it is highly likely that the benefit of ML might be greater in patients with less ideal acoustic windows.

Except for RAP and TRVmax, in our cohort we dealt with some degree of missing data. We expected the performance of the ML approaches to increase if more complete data were available for training. If we pre-impute and evaluate the performance, we achieve AUC values of 0.93. While this probably over-estimates the classification performance it gives reason to believe even better classification might be possible with more data. Interestingly, sensitivity is increased while specificity is diminished compared to Aduen et al. This can even be intensified by combining Aduen et al with ML. The higher specificity of Aduen et al. is, however, coupled with a lower negative predictive value, while for the proposed ML method the NPV and PPV are balanced. Although following the ML prediction more patients without PH will be subjected to RHC, we believe specificity is best warranted by RHC (rather than by echocardiography). RHC effectively limits the number of patients (without PH) that would falsely be subjected to therapy based on echocardiographic prediction, but by applying ML fewer patients with true PH will be excluded from RHC and hence from treatment and the confidence of rightly excluding these patients is higher compared to application of the formula by Aduen et al.

Our study has some limitations. The analysis focusses on distinguishing PH from no PH as defined via a binary cut-point at 25 mmHg, which does not consider borderline PH (20–24 mmHg), a simplification made due to the restrictive sample size. The foundation of ML projects remains high quality data acquisition and–annotation. This is why the dataset is limited to 90 patients from three institutions with a maximum interval of only 24h between echocardiography and the gold standard invasive measurement. Consequently, small sample size is a limitation of this cohort. Despite this restriction, our cohort is still the largest compared to previously published cohorts comparing echocardiographic to invasive determination and these studies applied less restrictive inclusion criteria—such as Chemla et al. (n = 31) [23], Friedberg et al. (n = 17) [24], Syyed et al (n = 65) [25], Dabestani et al. (n = 39) [26], Granstam et al (n = 29) [27], Kitabatake et al. (n = 33) [28]. On the other hand, the dataset is relatively small to train some ML models. However, ML has been applied very successfully in similar medical settings and sample sizes [22,29,30]. Moreover, the short delay (< 24 h) between echocardiography and invasive measurement in our cohort reduces the sample size, but adds to prediction accuracy, emphasizing that quality of annotation may compensate for small sample sizes in ML projects. The cohort only contains data from patients that allow calculation of Aduen et al. to allow for comparison against this algorithm. This raises the possibility that ML might perform better in a cohort with missing values for RAP or TR velocity. However, at this stage it is clear that no ML algorithm convincingly outperformed the simple Aduen formula and whether the conceptual advantages of ML algorithms may suffice to replace current approaches needs to be explored in a real world cohort that is larger by an order of a magnitude. Unfortunately, to the best of our knowledge such a cohort is currently unavailable.

In order to further counteract the limited size of the data set and the risk of overfitting our experimental setup, we used some expert knowledge: We binarized some variables using established cutoffs, we used pre-selected variables, and we removed patients with high degree of missing values. However, in comparison with the chosen ML algorithm, the other components of the experimental setup proved to be of little impact. For simulation purposes, we also included the continuous versions of the binarized variables, all variables instead of the filtered set, and all patients instead of only the ones with lower degrees of missing values. While the presented setup yields the best classification performance, the lowest AUC observed for the random forest of regression trees was 0.82 (95% CI 0.71–0.93), suggesting a high level of robustness. Nevertheless, although cross-validation compensates for the lack of a separate verification cohort, we still feel that prospective evaluation of the model in a large cohort should be the next step to evoke the expected paradigm shift in clinical decision making for PH. Of note, to date this has not been achieved for the suggested ESC algorithm and many traditional formulae.

Using ML approaches also provides a means to study and compare the importance of different variables–not only individually but their effect within multivariate modeling. We have seen that next to TRV, RVD2 is also highly informative. Thus, ML helps in understanding which of several parameters are associated with information gain in a particular setting. This also emerged from a recently conducted ML project with a large dataset, revealing important insights into prognostic performance of various echocardiographic variables [31]. When large cohorts are not available, our study demonstrates that ML is feasible to discriminate in smaller datasets. Thus ML may become a major component in clinical decision making in echocardiography in the near future [32].

## Conclusion

A late or missed diagnosis of PH may be detrimental. As ML algorithms can be easily integrated into echocardiographic machines, we explored the value of ML based statistics in the difficult clinical prediction of PH. The best machine learning algorithm for prediction of PH was equally accurate compared to the best traditional formula for estimating the likelihood of PH, already offering a reliable alternative with several conceptual advantages. The combination of both approaches further augmented the predictive accuracy and in particular sensitivity. Thus our ML algorithm may complement or replace the formula of Aduen et al. and may certainly replace it in cases were RAP cannot be determined reliably. Although the training data set is unique in terms of accuracy of PAP measurement, given the maximum of only 24 h between echocardiography and invasive measurement, the training set is small for ML. Thus provided our results can be confirmed in a larger independent cohort, the advantages of tolerance of missing values, its little reliance on RAP and competitive classification performance make our ML approach a smart alternative for prediction of the likelihood of PH.

## Supporting information

S1 Fig Correlation pairs. (PDF)

S2 Fig Clustering of variables based on correlation profiles. (PDF)

S3 Fig Setup of the cross validation. (PDF)

S4 Fig ROC curves. (PDF)

S5 Fig Classification performance assessed by precision recall (PR) curves. (PDF)

S6 Fig Bland-Altman plots. (PDF)

S7 Fig Analysis of variable importance for all ML algorithms. (PDF)

S1 Appendix Supplementary methods and references, TRIPOD checklist and supplementary figure legends. (PDF)

## Abbreviations

ACC: accuracy
AR: aortic regurgitation
AS: aortic stenosis
AUC: area under the ROC curve
CV: cross validation
ESC: European Society of Cardiology
FAMD: factor analysis for mixed data
ML: machine learning
MR: mitral regurgitation
MS: mitral stenosis
NPV: negative predictive value
PAP: pulmonary artery pressure
PAPm: mean pulmonary artery pressure
PAPsys: systolic pulmonary artery pressure
PAT: pulmonary (valve) acceleration time
PH: pulmonary hypertension
PPV: positive predictive value
RA: right atrium
RAP: right atrial pressure
RHC: right heart catheterization
ROC: receiver operator characteristic
RV: right ventricular
RVD: right ventricular diameter
SVM: support vector machine
TAPSE: tricuspid annular plane systolic excursion
TR: tricuspid regurgitation
TRPm: RV-RA mean gradient
TRV: tricuspid regurgitation velocity
TRVmax: tricuspid regurgitation maximal velocity
TS: tricuspid stenosis
