# Supplementary figures and images for: A machine learning approach for the prediction of pulmonary hypertension
Source: PLoS One. 2019 Oct 25;14(10):e0224453. doi: 10.1371/journal.pone.0224453 (PMC6814224; doi:10.1371/journal.pone.0224453)

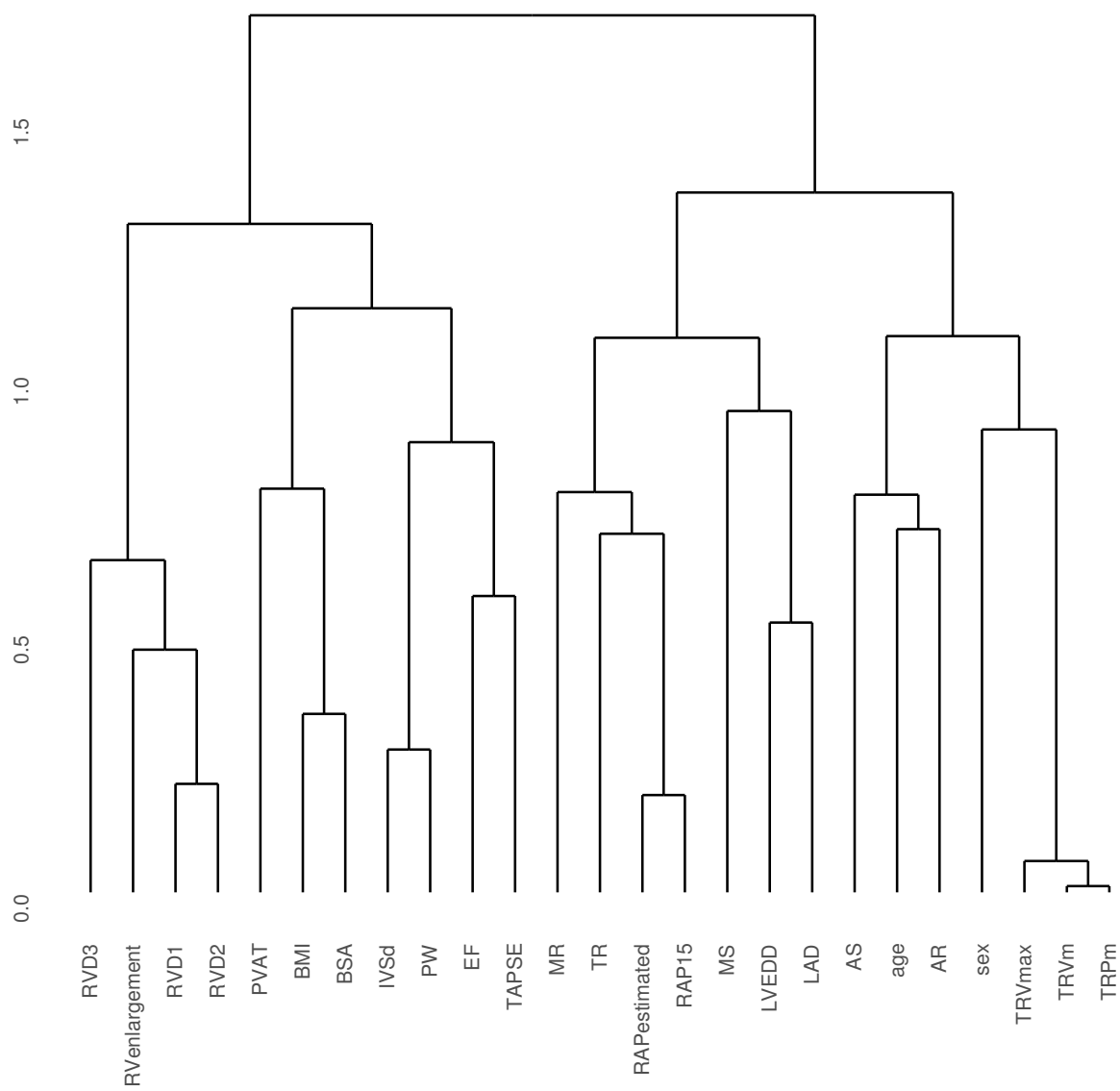

Supplement: S2 Fig — (PDF) [file pone.0224453.s002.pdf]

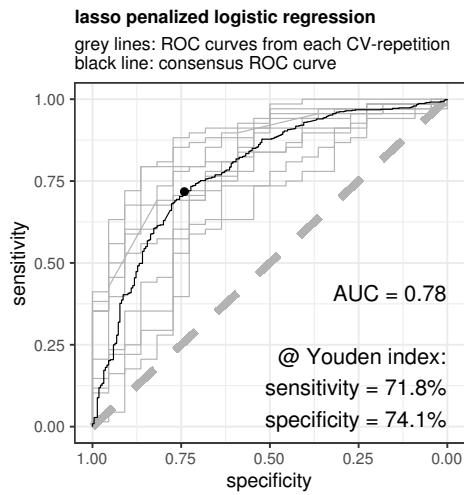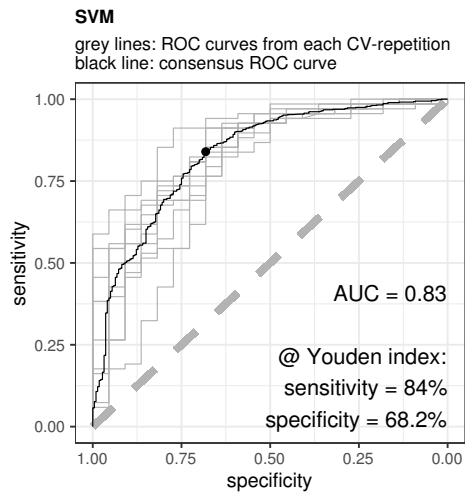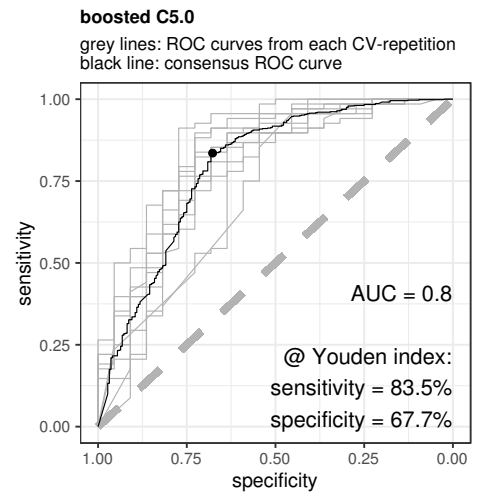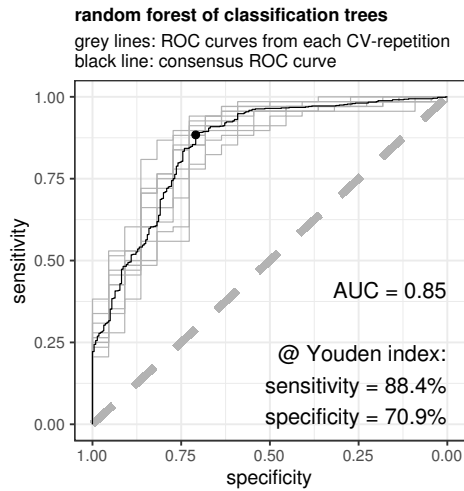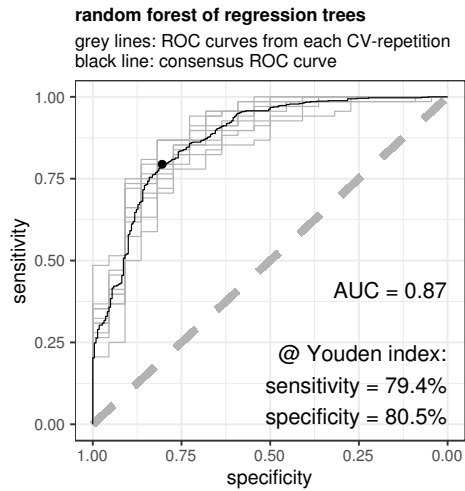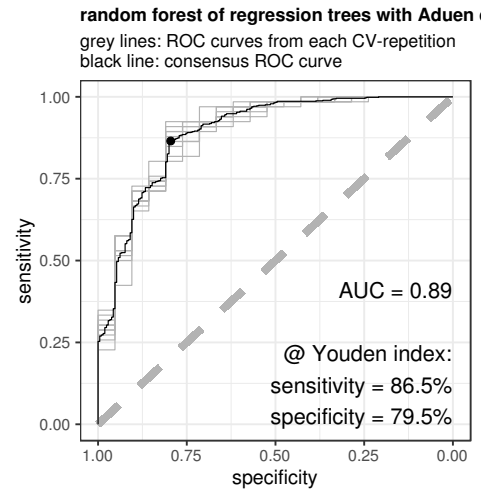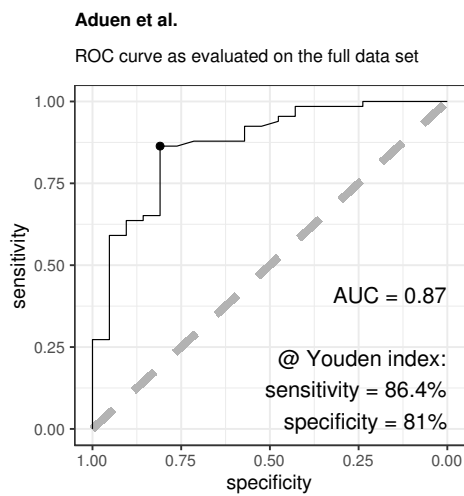

Supplement: S4 Fig — (PDF) [file pone.0224453.s004.pdf]

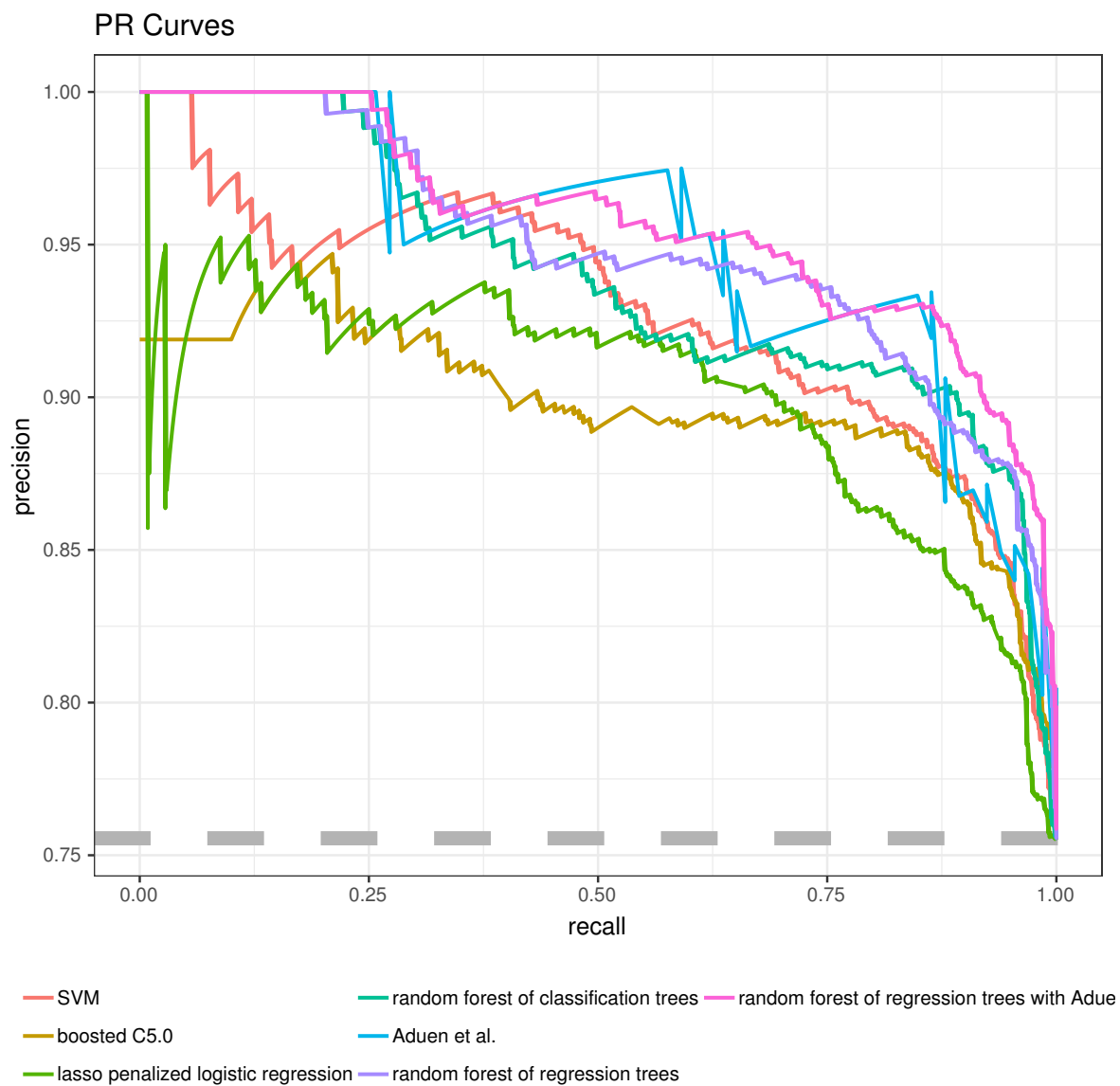

Supplement: S5 Fig — (PDF) [file pone.0224453.s005.pdf]
