# Supplementary material for: A machine learning approach for the prediction of pulmonary hypertension
Source: PLoS One. 2019 Oct 25;14(10):e0224453. doi: 10.1371/journal.pone.0224453 (PMC6814224; doi:10.1371/journal.pone.0224453)
Supplement: S6 Fig — (PDF) [file pone.0224453.s006.pdf]

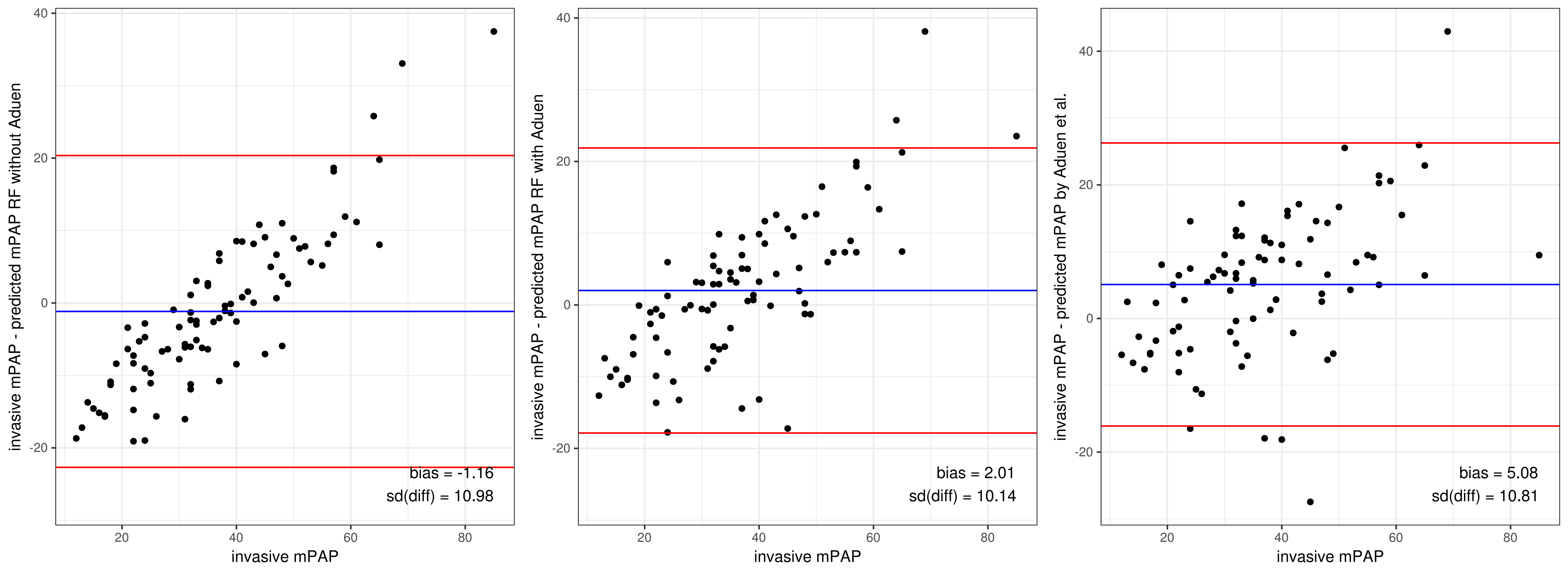

invasive mPAP - predicted mPAP RF without Aduen
bias = -1.16
sd(diff) = 10.98
invasive mPAP
invasive mPAP - predicted mPAP RF with Aduen
bias = 2.01
sd(diff) = 10.14
invasive mPAP
invasive mPAP - predicted mPAP by Aduen et al.
bias = 5.08
sd(diff) = 10.81
invasive mPAP
