# Supplementary material for: A machine learning approach for the prediction of pulmonary hypertension
Source: PLoS One. 2019 Oct 25;14(10):e0224453. doi: 10.1371/journal.pone.0224453 (PMC6814224; doi:10.1371/journal.pone.0224453)
Supplement: S7 Fig — (PDF) [file pone.0224453.s007.pdf]

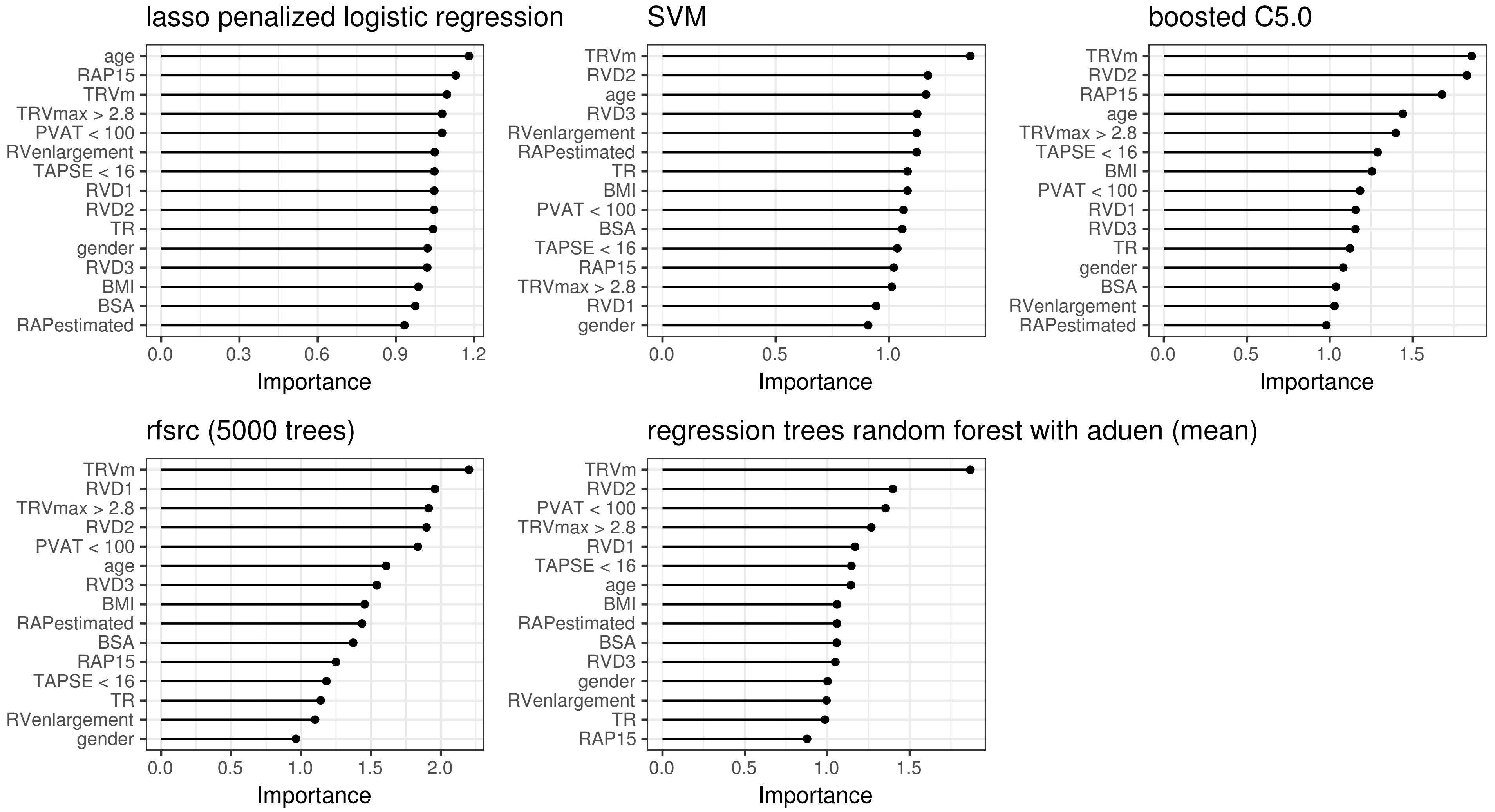
lasso penalized logistic regression
age
RAP15
TRVm
TRVmax > 2.8
PVAT < 100
RVenlargement
TAPSE < 16
RVD1
RVD2
TR
gender
RVD3
BMI
BSA
RAPestimated
0.0 0.3 0.6 0.9 1.2
Importance
SVM
TRVm
RVD2
age
RVD3
RVenlargement
RAPestimated
TR
BMI
PVAT < 100
BSA
TAPSE < 16
RAP15
TRVmax > 2.8
RVD1
gender
0.0 0.5 1.0
Importance
boosted C5.0
TRVm
RVD2
RAP15
age
TRVmax > 2.8
TAPSE < 16
BMI
PVAT < 100
RVD1
RVD3
TR
gender
BSA
RVenlargement
RAPestimated
0.0 0.5 1.0 1.5
Importance
rfsrc (5000 trees)
TRVm
RVD1
TRVmax > 2.8
RVD2
PVAT < 100
age
RVD3
BMI
RAPestimated
BSA
RAP15
TAPSE < 16
TR
RVenlargement
gender
0.0 0.5 1.0 1.5 2.0
Importance
regression trees random forest with aduen (mean)
TRVm
RVD2
PVAT < 100
TRVmax > 2.8
RVD1
TAPSE < 16
age
BMI
RAPestimated
BSA
RVD3
gender
RVenlargement
TR
RAP15
0.0 0.5 1.0 1.5
Importance
