# Supplementary material for: A machine learning approach for the prediction of pulmonary hypertension
Source: PLoS One. 2019 Oct 25;14(10):e0224453. doi: 10.1371/journal.pone.0224453 (PMC6814224; doi:10.1371/journal.pone.0224453)
Supplement: S1 Fig — (PDF) [file pone.0224453.s001.pdf]

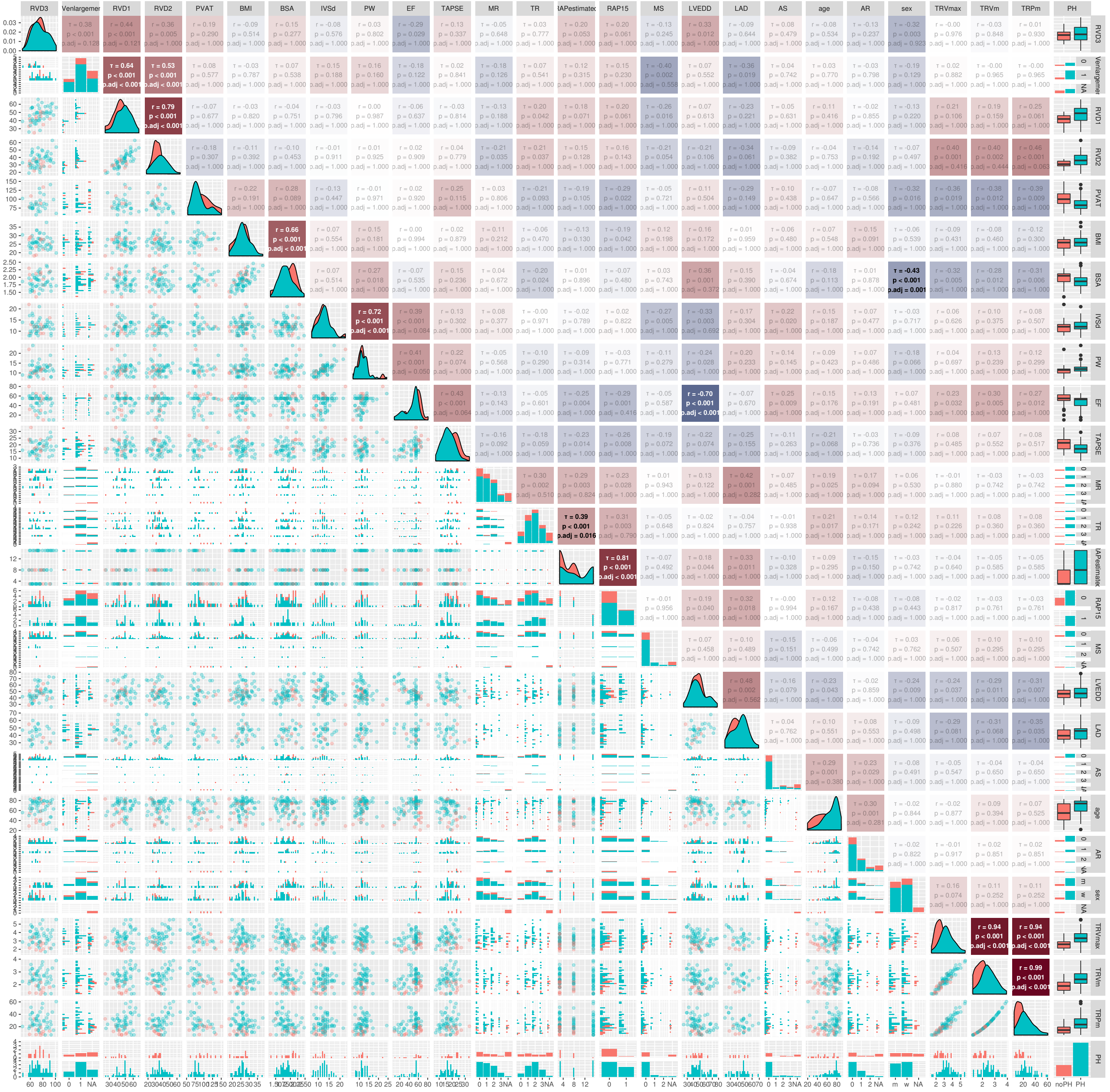

supplementary figure 1
