# Supplementary material for: A machine learning approach for the prediction of pulmonary hypertension
Source: PLoS One. 2019 Oct 25;14(10):e0224453. doi: 10.1371/journal.pone.0224453 (PMC6814224; doi:10.1371/journal.pone.0224453)
Supplement: S1 Appendix — (PDF) [file pone.0224453.s008.pdf]

S1 – Appendix to

A machine learning approach for the prediction of pulmonary hypertension

Andreas Leha, Kristian Hellenkamp, Bernhard Unsöld, Sitali Mushemi-Blake, Ajay M. Shah, Gerd Hasenfuß, Tim Seidler

Table of Contents

- Supplementary Methods 2-4
- TRIPOD checklist 5-6
- Supplementary References 7
- Supplementary Figure Legends 8-10

Supplementary Methods

Technical Details for the Machine Learning Algorithms

The SVMs were trained for classification using a radial kernel. The data were scaled to zero mean and unit variance prior to training. The hyperparameters 'cost' and γ were set using an internal 10-fold cross validation. The implementation in the R package e1071 version 1.6-8 [1] was used to train the SVMs.

The R package C50 [2] was used in version 0.1.1 for the boosted classification tree models. Up to 100 iterations of boosting were allowed. The default options were used: Winnowing was not applied, global pruning was enabled, the confidence factor was set to 0.25, a minimum of 2 cases was required, fuzzy thresholds were not used, and early stopping was allowed.

Both random forest models were trained through the R package randomForestSRC ([3], version 2.5.1) and 5000 trees were grown.

Using as case weights the inverse prevalence the class imbalance was handled in the random forest of classification trees. Splitting was done using the Gini index splitting ([4], Chapter 4.3).

The regression trees were trained to predict the invasively measured PAPm. As splitting rule the weighted mean-squared error splitting was used ([4], Chapter 8.4). The class prediction (PH vs noPH) was then done using the value corresponding to the Youden index as cutoff.

All other parameters for the random forest models were left at their default values: boostrap resamples of the size of 0.632 of the sample size in the training data were drawn with replacement at the root node, the (rounded) square root of the number of available features was sampled in each split, the forest average number of unique cases (data points) in a terminal node was set to 1, deterministic splitting was used,

The hyperparameter λ in the lasso penalized logistic regression model was chosen using an internal 10-fold cross validation. The R package glmnet was facilitated in version 2.0.16. The

alpha parameter was fixed at '1' to arrive at lasso penalization. The other parameters were used at their default values: The data was standardized, intercepts were included in the models, uniform case weights were used, no offsets were given.

Technical Details for the Statistical Analysis

As descriptive values continuous variables have been summarized by mean and standard deviation as well as median, minimum and maximum. Categorical variables are summarized with absolute and relative frequencies. Pairwise correlations were calculated and tested for significance using Pearson's correlation coefficient and Kendall's τ as appropriate. P values were adjusted for multiplicity using Holm's procedure. Hierarchical clustering was applied on the pairwise correlation profiles.

For the visualization of the data facilitating dimension reduction by factor analysis for mixed data (FAMD) imputation based on FAMD on the full data set was applied. Prior to that and only for the visualization variables were scaled, the data (both, variables and patients) hierarchically clustered and presented as a heatmap.

Prior to applying the lasso penalized logistic regression and the SVM missing values were imputed using the iterative FAMD algorithm. In short, the first step is to recode categorical variables by dummy variables. The imputation starts with an initial simple mean imputation which is iteratively replaced by FAMD reconstructions.

Variable importance for the regression tree forest was calculated using Breiman-Cutler permutation variable importance. In short, variable importance is measured by the average (across all trees) increase in the out-of-bag prediction error when the variable is randomly permuted. The reported variable importance was averaged across the CV repeats.

The predictions of the PAPm measurement from random forest of regression trees have additionally been combined – by taking the mean – with the predictions obtained through the formula by Aduen et al. (called *random forest of regression trees with Aduen et al.*).
Several conventional formulae have been suggested to estimate PAP or the likelihood of PH. For comparison, the best of several formulae (that by Aduen et al.) as systematically compared in [5], has been included as reference.

Individual ROC curves for each repetition of the CV are presented as well as a consensus ROC curve pooled over all repetitions. The Youden index was determined. Sensitivity, specificity, positive predictive value (PPV), negative predictive value (NPV), and accuracy were evaluated at the Youden index. For regression tree random forest, the Pearson's correlation coefficient of measured and predicted PAPm values together with its 95% confidence interval were calculated. Average values across CV repetitions are reported.

Tripod Checklist

The guidelines of the transparent reporting of a multivariable prediction model for individual prognosis or diagnosis (TRIPOD) statement [6] were followed.

Section/Topic	Ite		Checklist Item	Page
**Title and abstract**				
Title	1	D;V	Identify the study as developing and/or validating a multivariable prediction model, the target population, and the outcome to be predicted.	
Abstract	2	D;V	Provide a summary of objectives, study design, setting, participants, sample size, predictors, outcome, statistical analysis, results, and conclusions.	2
**Introduction**				
Background and objectives	3a	D;V	Explain the medical context (including whether diagnostic or prognostic) and rationale for developing or validating the multivariable prediction model, including references to existing models.	7
	3b	D;V	Specify the objectives, including whether the study describes the development or validation of the model or both.	8
**Methods**				
Source of data	4a	D;V	Describe the study design or source of data (e.g., randomized trial, cohort, or registry data), separately for the development and validation data sets, if applicable.	9
	4b	D;V	Specify the key study dates, including start of accrual; end of accrual; and, if applicable, end of follow-up.	9
Participants	5a	D;V	Specify key elements of the study setting (e.g., primary care, secondary care, general population) including number and location of centres.	9
	5b	D;V	Describe eligibility criteria for participants.	9
	5c	D;V	Give details of treatments received, if relevant.	n/a
Outcome	6a	D;V	Clearly define the outcome that is predicted by the prediction model, including how and when assessed.	10
	6b	D;V	Report any actions to blind assessment of the outcome to be predicted.	n/a
Predictors	7a	D;V	Clearly define all predictors used in developing or validating the multivariable prediction model, including how and when they were measured.	9+Tab1
	7b	D;V	Report any actions to blind assessment of predictors for the outcome and other predictors.	n/a
Sample size	8	D;V	Explain how the study size was arrived at.	9
Missing data	9	D;V	Describe how missing data were handled (e.g., complete-case analysis, single imputation, multiple imputation) with details of any imputation method.	11
Statistical analysis methods	10a	D	Describe how predictors were handled in the analyses.	10
	10b	D	Specify type of model, all model-building procedures (including any predictor selection), and method for internal validation.	10
	10c	V	For validation, describe how the predictions were calculated.	n/a
	10d	D;V	Specify all measures used to assess model performance and, if relevant, to compare multiple models.	11
	10e	V	Describe any model updating (e.g., recalibration) arising from the validation, if done.	n/a
Risk groups	11	D;V	Provide details on how risk groups were created, if done.	n/a
Development vs. validation	12	V	For validation, identify any differences from the development data in setting, eligibility criteria, outcome, and predictors.	n/a
**Results**				
Participants	13a	D;V	Describe the flow of participants through the study, including the number of participants with and without the outcome and, if applicable, a summary of the follow-up time. A diagram may be helpful.	Fig 4
	13b	D;V	Describe the characteristics of the participants (basic demographics, clinical features, available predictors), including the number of participants with missing data for predictors and outcome.	Tab 1
	13c	V	For validation, show a comparison with the development data of the distribution of important variables (demographics, predictors and outcome).	n/a
Model development	14a	D	Specify the number of participants and outcome events in each analysis.	Tab1
	14b	D	If done, report the unadjusted association between each candidate predictor and outcome.	Tab1
Model specification	15a	D	Present the full prediction model to allow predictions for individuals (i.e., all regression coefficients, and model intercept or baseline survival at a given time point).	n/a
	15b	D	Explain how to the use the prediction model.	16
Model performance	16	D;V	Report performance measures (with CIs) for the prediction model.	Tab2

Model-updating	17	V	If done, report the results from any model updating (i.e., model specification, model performance).	n/a
**Discussion**				
Limitations	18	D;V	Discuss any limitations of the study (such as nonrepresentative sample, few events per predictor, missing data).	17 f.
Interpretation	19a	V	For validation, discuss the results with reference to performance in the development data, and any other validation data.	n/a
	19b	D;V	Give an overall interpretation of the results, considering objectives, limitations, results from similar studies, and other relevant evidence.	19
Implications	20	D;V	Discuss the potential clinical use of the model and implications for future research.	19
**Other information**				
Supplementary information	21	D;V	Provide information about the availability of supplementary resources, such as study protocol, Web calculator, and data sets.	20
Funding	22	D;V	Give the source of funding and the role of the funders for the present study.	20

*Items relevant only to the development of a prediction model are denoted by D, items relating solely to a validation of a prediction model are denoted by V, and items relating to both are denoted D;V. We recommend using the TRIPOD Checklist in conjunction with the TRIPOD Explanation and Elaboration document.

Supplementary References

1. Meyer D et al. e1071: Misc Functions of the Department of Statistics, Probability Theory Group (Formerly: E1071), TU Wien. 2017. link.

2. Kuhn M and Quinlan R. C50: C5.0 Decision Trees and Rule-Based Models. 2017. link.

3. Ishwaran H and Kogalur UB. Random Forests for Survival, Regression, and Classification (RF-SRC). 2017. link.

4. Breiman L et al. Classification and Regression Trees. Chapman and Hall/CRC 1984.

5. Hellenkamp K, Unsold B, Mushemi-Blake S, Shah AM, Friede T, Hasenfuss G, et al. Echocardiographic Estimation of Mean Pulmonary Artery Pressure: A Comparison of Different Approaches to Assign the Likelihood of Pulmonary Hypertension. Journal of the American Society of Echocardiography : official publication of the American Society of Echocardiography. 2018 Jan;31(1):89-98.

6. Gary S. Collins, Johannes B. Reitsma, Douglas G. Altman, Karel G.M. Moons; on behalf of the TRIPOD Group. Transparent Reporting of a multivariable prediction model for Individual Prognosis Or Diagnosis (TRIPOD): The TRIPOD Statement. Ann intern Med 2015; 162: 55-63.

Supplementary Figure Legends

S1 Fig. Correlation pairs. Not intended for printout in letter format - to be viewed on a larger screen for expansion.

All pairwise correlations between the variables have been calculated. Kendall's τ is used for discrete variables, Pearson's correlation otherwise. Each correlation is tested for significance. The resulting p values are adjusted for multiplicity using Holm's procedure. The upper triangle shows the results as a heatmap colored by the correlation coefficient: blue shades represent negative correlations, red shades represent positive correlations. Bold text shows correlations which remain significant after multiplicity adjustment. The panels on the diagonal show the distribution of the variables grouped by PH. Barplots are shown for discrete variables, density plots otherwise. The lower triangle visualizes pairs of variables coloured by PH. Scatter plots are used if both variables are continuous, histograms if only one variable is continuous, and barplots if both variables are discrete. The variables are ordered using hierarchical clustering on their correlation profiles. (Not intended for printout in letter format - to be viewed on a larger screen for expansion).

S2 Fig. Clustering of variables based on correlation profiles. Variables are pairwise correlated. This dendrogram shows a clustering based on the profiles of these pairwise correlations.

S3 Fig. Setup of the cross validation. A stratified 10 times repeated 3 fold cross validation scheme was used to assess the classification performance. Panel (A) shows the number of in the test set of each fold. Panel (B) shows the allocation of patients to training and test set in each fold. Panel (C) shows the distribution of PH (named '1') and no PH (named '0') in each

fold. As the cross validation is stratified the distribution in each training and test set is similar. (Not intended for printout in letter format - to be viewed on a larger screen for expansion).

S4 Fig. ROC Curves. For the five machine learning algorithms under consideration gray lines show the ROC curves from the single repeats of the cross validation and the black line shows the consensus ROC curve across all repeats. For the established method by Aduen et al. the ROC curve from the prediction of the full data set is shown. The black dot mark the Youden index. The text gives the AUC and the sensitivity/specificity.

S5 Fig. Classification performance assessed by precision recall (PR) curves. Random forest of regression trees shows performance comparable to the best of several established PH prediction methods by Aduen et al. Consensus PR curves for the prediction of PH of the five machine learning algorithms under consideration as well as the PR curve of the method by Aduen et al. (light blue).

S6 Fig. Bland-Altman-Plots. The difference between the prediction of the mPAP and the invasively determined mPAP (y axis) are plotted against the invasively determined mPAP (x axis). The left panel shows the predictions by random forest of regression trees, the center panel shows the predictions of the combination of Aduen et al. with the random forest of regression trees, the right panel shows the predictions of Aduen et al.. The results of the first repetition of the CV are displayed in the left and center panel. While the bias increases towards prediction without ML (Aduen et al. on average predict 5mmHG to low), the variance of the difference stays nearly constant. In the ML approach the regression to the mean is clearly visible as trend in the Bland-Altman-Plot (left and center panels).

S7 Fig. Analysis of variable importance for all ML algorithms. This plot shows the variable importance measures for the remaining ML methods besides the top-performing random forest of regression trees (main text, Figure 3 (B)). Shown is the increase in prediction error after permuting the variable in question. For classification methods the prediction error is measured as the log-loss, for the regression trees random forest with Aduen the prediction error is measured as mean squared error. The regression model in the lasso penalized logistic regression does not include any interaction terms, so the lasso penalized logistic regression yields expectedly different results from the other methods. All other methods rank TRVm as most important and have RVD2 among the most important variables. As the random forest of regression trees, the other methods based on random forest, rfsrc and regression trees random forest with Aduen, attribute less importance to the RAP variables.
